# Commentary: Superoxide Generation and Its Involvement in the Growth of *Mycobacterium smegmatis*

**DOI:** 10.3389/fmicb.2017.01114

**Published:** 2017-06-13

**Authors:** Abhishek Mishra

**Affiliations:** Department of Biotechnology, Goa University, Taleigao PlateauPanaji, India

**Keywords:** dihydroethidium, HPLC, superoxide, PEG-SOD, NADH oxidase, *Mycobacterium smegmatis*

In a recent publication authors claim that a continuous generation of superoxide via NADH oxidase is essential for the growth of *Mycobacterium smegmatis* (Yeware et al., 2017). The major issue with this report is extremely high superoxide estimation which is contrary to a previous study that records a negligible production of superoxide in *M. smegmatis* cells using the same method (Tyagi et al., 2015). To address this controversy, I did a careful investigation and concluded that dihydroethidium (DHE)-HPLC profiles were misrepresented which has led to an overestimation of superoxide production. Therefore, I present the case that the authors have failed to show evidence for superoxide generation from NADH oxidase/cell and presumed its physiological role in growth of *M. smegmatis*. This conclusion is supported by following reasons.

Stoichiometric ratio of superoxide and oxyethidium formation is 1:1 in superoxide reaction with DHE (Zielonka et al., 2008). Yeware et al. (2017), showed ~8 μM 2-OH-E^+^ per 15 μg of cell protein in 30 min which translate into ~0.5 mM 2-OH-E^+^ per mg protein, which is extremely high concentration, almost 1,000 times more in comparison to the maximum reported in biological system. In general physiological consequence of such high concentration should be deleterious as it is a potent disrupter of biomolecules. To date superoxide estimation in living organism was found to be in the range of 10^−9^–10^−12^ mole/mg of protein. In cells and tissues, DHE reacts specifically with superoxide to form 2-OH-E^+^, whereas other cellular components non-specifically react with DHE to form E^+^. As a result E^+^ is produced in much higher concentration than 2-OH-E^+^ in biological systems (Zielonka et al., 2008; Kalyanaraman et al., 2014). Based on previous reports, Table 1 represents the approximate ratio of 2-OH-E^+^ and E^+^ to emphasize the higher concentration E^+^ among the two in biological samples. Whereas, the HPLC profile presented by Yeware et al., shows a tiny peak of E^+^ which is negligibly smaller than 2-OH-E^+^ on an arbitrary scale. Furthermore, authors observed constant increase in 2-OH-E^+^ but no significant change in E^+^ for the period of 3 h in their study. This discrepancy could have been avoided if authors ensured that some detectable amount of unoxidized DHE remain in their assay while standarizing the balance between cell number and incubation time with dye. Importantly, the authors should present standard chromatographic profile of DHE, 2-OH-E^+^, and E^+^ with positive (menadione or pyrogallol) and negative control (SOD or TEMPOL) to justify the exceptional HPLC profile obtained in the experiment.

**Table 1:** Relative detection of 2-OH-E^+^ and E^+^ in biological samples.

**Cells/Tissue/Cell lysate**	2-OH-E^+^:E^+^ ratio^*^	**References**
Murine macrophages (RAW 264.7)	1:10	Zielonka et al., 2008
Vascular smooth muscle cells	1:1.8	Fernandes et al., 2007
Vascular smooth muscle cells + Angiotensin II	1:1.2	
Lung epithelial cells lysate + NADH	1:3	Gray et al., 2007
Lung epithelial cells lysate + NADH + Paraquat	1:1.25	
Bovine arotic endothelial cells	1:10	Zhao et al., 2005
Bovine arotic endothelial cells + Menadione (5 μM)	1:6	
Bovine arotic endothelial cells + Menadione (20 μM)	1:2	
Bovine arotic endothelial cells + Angiotensin II	1:3	Laurindo et al., 2008
Bovine arotic endothelial cell lysate	1:80	Zielonka et al., 2009
Dopaminergic N27 cell line	1:50	Dranka et al., 2012
Dopaminergic N27 cell line + Rotenone	1:100	
Dopaminergic N27 cell line + 6-hydroxydopamine	1:50	
Dopaminergic N27 cell line + Paraquat	1:20	
Dopaminergic N27 cell line + Menadione	1:10	
Dopaminergic N27 cell line + 1-methyl-4-phenylpyridinium	1:20	
Spermatozoa	1:2.5	De Iuliis et al., 2006
Spermatozoa + Menadione	1.5:1	
Human neuroblastoma cells	1:2	Shang et al., 2005
Human neuroblastoma cells + 1-methyl-4-phenylpyridinium	1:1	

* *Approximate ratio is calculated from the data reported in references*.

SOD is a standard negative control for the detection of superoxide due to the fact that it reacts with superoxide at 1,000 times higher rate in comparison to DHE (Zielonka et al., 2008). Yeware et al. (2017), observed no effect of PEG-SOD on either growth or superoxide production in cells, which they interpret as non-permeability of PEG-SOD. Moreover, in crude membrane preparation PEG-SOD could barely inhibit 0.37 folds of 2-OH-E^+^. Another important superoxide scavenger used in this study is membrane permeable SOD mimetic TEMPOL (4-hydroxy-Tempo). Although reaction rate constant of TEMPOL with superoxide is much lower than that of SOD it has a similar *in vivo* efficacy which is ascribed to its smaller size and higher pentrability (Luo et al., 2009). However, unlike SOD, TEMPOL can non-specifically react with other reactive oxygen species like hydrogen peroxide or hydroxyl radical. Contrary to their claim, authors report TEMPOL was able to inhibit superoxide production but has no effect on growth of *M. smegmatis* even at 10 times higher concentration. This leads to an ambiguous conclusion that superoxide generation inhibited by diphenyliodonium (DPI), but not by TEMPOL, has a role in growth. Noteworthy, DPI is a non-specific inhibitor of flavoenzymes, in addition, it inhibits pentose phosphate pathway and tricarboxylic acid cycle (Riganti et al., 2004). Therefore, superoxide lowering effect of DPI *in vivo* could have been observed due to reduced derivation of both, 2-OH-E^+^ and E^+^. Obviously, no effect of rotenone and antimycin A was observed as they are inhibitor of mitochondrial electron transport chain (ETC) and generally ineffective against bacteria. In particular, mycobacterial ETC is more complex than mitochondrial ETC due to diversity of components and branching (Cook et al., 2004). Surprisingly, authors have not used any of the standard mycobacterial ETC inhibitor (Black et al., 2014).

DHE-HPLC assay needs a careful standardization and should be supported by another standard method of superoxide estimation to affirm its biological role (Forman et al., 2015). Particularly in this case author should have been able to measure microMolar concentration of superoxide, as per their claim, by gold standard cytochrome C reduction assay. The purpose of the present communication is to ensure that the inappropriate quantitative estimation of superoxide generation do not form the basis for further erroneous papers. Moreover, to highlight the general aspects of experimental design and considerations for the measurement of superoxide radical in biological samples.

## Author contributions

The author confirms being the sole contributor of this work and approved it for publication.

### Conflict of interest statement

The author declare that the research was conducted in the absence of any commercial or financial relationships that could be construed as a potential conflict of interest.
